# Individualized Assays of Temporal Coding in the Ascending Human Auditory System

**DOI:** 10.1523/ENEURO.0378-21.2022

**Published:** 2022-03-11

**Authors:** Agudemu Borjigin, Alexandra R. Hustedt-Mai, Hari M. Bharadwaj

**Affiliations:** 1 Weldon School of Biomedical Engineering, Purdue University, West Lafayette, IN; 2 Department of Speech, Language, and Hearing Sciences, Purdue University, West Lafayette, IN 47907

**Keywords:** electroencephalography, frequency modulation, interaural time difference, neural coding, nonsensory factors, temporal fine structure

## Abstract

Neural phase-locking to temporal fluctuations is a fundamental and unique mechanism by which acoustic information is encoded by the auditory system. The perceptual role of this metabolically expensive mechanism, the neural phase-locking to temporal fine structure (TFS) in particular, is debated. Although hypothesized, it is unclear whether auditory perceptual deficits in certain clinical populations are attributable to deficits in TFS coding. Efforts to uncover the role of TFS have been impeded by the fact that there are no established assays for quantifying the fidelity of TFS coding at the individual level. While many candidates have been proposed, for an assay to be useful, it should not only intrinsically depend on TFS coding, but should also have the property that individual differences in the assay reflect TFS coding per se over and beyond other sources of variance. Here, we evaluate a range of behavioral and electroencephalogram (EEG)-based measures as candidate individualized measures of TFS sensitivity. Our comparisons of behavioral and EEG-based metrics suggest that extraneous variables dominate both behavioral scores and EEG amplitude metrics, rendering them ineffective. After adjusting behavioral scores using lapse rates, and extracting latency or percent-growth metrics from EEG, interaural timing sensitivity measures exhibit robust behavior-EEG correlations. Together with the fact that unambiguous theoretical links can be made relating binaural measures and phase-locking to TFS, our results suggest that these “adjusted” binaural assays may be well suited for quantifying individual TFS processing.

## Significance Statement

The auditory system is unique among the senses in that neurons in the periphery fire precisely phase-locked spikes in response to fast temporal fluctuations in sound. Yet, the functional significance of this metabolically expensive initial neural code is debated. Here, we establish behavioral and physiological assays that can probe the fidelity of this phase-locking mechanism at the level of individual human subjects. These measures pave the way for future experiments that can more directly address foundational questions about the role of phase locking in everyday hearing, and test whether phase-locking deficits contribute to the listening difficulties in clinical populations. Importantly, our results also show that commonly used measures to assess phase locking are affected by extraneous variables, and thus ineffective.

## Introduction

All acoustic information we receive is conveyed through the firing rate and/or timing of the neural spikes (i.e., rate-place vs temporal coding) of cochlear neurons. Temporal information in the basilar-membrane vibrations consists of cycle-by-cycle variations in phase, the temporal fine structure (TFS), and dynamic variations in amplitude, the envelope (ENV; Hilbert, 1906). Cochlear neurons phase-lock to both TFS (Johnson, 1980), and ENV (Joris and Yin, 1992) robustly, with TFS phase-locking extending at least up to 1000 Hz (Verschooten et al., 2019). While the peripheral rate-place code has consistent counterparts throughout the auditory system, the upper limit of phase-locking progressively shifts to lower frequencies along the ascending pathway (Joris et al., 2004). How this metabolically expensive initial/peripheral temporal code (Laughlin et al., 1998; Hasenstaub et al., 2010) contributes to everyday hearing and how its degradation contributes to perceptual deficits are foundational questions in auditory neuroscience and clinical audiology. Yet, the significance of TFS coding is debated (Drullman, 1995; Oxenham and Simonson, 2009; Swaminathan and Heinz, 2012; Oxenham, 2013).

Previous studies have explored whether sound localization and pitch perception benefit from TFS cues. While it is established that lateralization of low-frequency sounds depends on TFS (Smith et al., 2002; Yin and Chan, 1990), whether TFS is important for pitch perception is difficult to ascertain. Behavioral studies suggest that low-frequency periodic sounds elicit a stronger pitch than high-frequency sounds (Moore, 1973; Houtsma and Smurzynski, 1990; Bernstein and Oxenham, 2003), suggesting a possible role for TFS. However, these results permit alternate interpretations in terms of place coding and harmonic resolvability (Oxenham, 2012). Regardless of its role in quiet, whether TFS is important for masking release in noise is further debated, especially when other redundant cues can also convey pitch or location, and when room reverberation can degrade temporal cues (Best et al., 2005; Oxenham and Simonson, 2009; Ihlefeld and Shinn-Cunningham, 2011).

To investigate the role of TFS, studies have used sub-band vocoding to independently manipulate ENV and TFS cues (Smith et al., 2002; Hopkins et al., 2008; Hopkins and Moore, 2009; Lorenzi et al., 2009; Ardoint and Lorenzi, 2010). However, acoustic manipulations cannot eliminate subsequent confounding of ENV, TFS, and place cues without detailed knowledge of cochlear processing at the individual level (Swaminathan and Heinz, 2012; Oxenham, 2013). Thus, establishing the precise role of TFS through vocoding experiments is difficult, although the use of high-fidelity vocoders can help (Viswanathan et al., 2021). An alternative approach is to directly measure TFS sensitivity from individual listeners and compare it to individual differences in other perceptual measures. The individual-differences approach has been successfully used to address other fundamental questions (McDermott et al., 2010; Bharadwaj et al., 2015; Whiteford et al., 2020). Unfortunately, the lack of established measures of TFS sensitivity at the individual level limits this enterprise.

Conventional behavioral TFS-sensitivity measurements have attempted to eliminate confounding cues such that primary task would rely on TFS processing (Strelcyk and Dau, 2009; Moore and Sek, 2009; Hopkins and Moore, 2010; Sek and Moore, 2012). However, they did not assess the influence of extraneous factors on the measured scores. Unfortunately, nonsensory factors can contribute significantly to individual variability even when the tasks themselves rely on specific acoustic cues (Kidd et al., 2007). Objective electrophysiological measures of TFS sensitivity can circumnavigate this problem; however, such studies are scarce (Verschooten et al., 2015; Parthasarathy et al., 2020). Here, we employ a battery of both behavioral and electroencephalography (EEG)-based measures of TFS sensitivity on a cohort of normal-hearing (NH) individuals to identify candidate assays of TFS processing at the individual level. Our results suggest that extraneous variables dominate both behavioral and raw EEG measures. However, with adjustments, we observed robust behavior-EEG correlations in binaural assays, rendering them well suited for quantifying individual TFS processing.

## Materials and Methods

The primary goal of the current study was to evaluate an array of both behavioral and electrophysiological measures as candidate assays of TFS sensitivity at the individual level. Based on the finding that nonsensory factors contribute significantly to behavioral TFS measures, a large-N supplementary behavioral experiment was conducted to assess whether nonsensory factors also influence ENV sensitivity when measured from naive participants.

### Participants

One hundred and fifty-three listeners, aged 18–60 years, were recruited from the local community near Purdue University. All human subject measures were conducted following protocols approved by the Purdue University Internal Review Board and the Human Research Protection Program. Participants were recruited via posted flyers and bulletin-board advertisements and provided informed consent. All participants had pure-tone air-conduction thresholds of 25 dB Hearing Level (HL) or better at octave frequencies from 500 to 8000 Hz. Of the 153 subjects, 44 (20 males) participated in the main experiments designed to evaluate candidate assays of TFS processing. The remaining *N* = 109 participated in the supplementary experiment aimed at testing whether nonsensory factors also influence ENV sensitivity. Although the goal of the main experiment was to conduct all behavioral and electrophysiological TFS measures on each participant, some were not able to finish the full study battery because of limited availability. Among the 44 listeners who participated in the main study, 43 completed the frequency modulation (FM) detection task, and 36 completed the interaural time difference (ITD) detection task. The intersection, 33 subjects, completed both behavioral measurements. Among all participants (*n* = 44), 42 subjects completed EEG-ITD sensitivity measurements; 25 of those 42 subjects also completed EEG-frequency following response (FFR) measurements. Among the subjects who completed both behavioral measurements, all except one (*n* = 32) completed the EEG-ITD measurement; these subjects include all participants who completed the EEG-FFR measurements (*n* = 25). The subjects who completed both behavioral measurements (*n* = 32, age: mean = 26.8, SD = 11.2) were included for the main analyses including brain-behavior correlations. Although the age range was wide, only six out of 33 subjects were older than 35 years at the time of the testing, and age did not significantly correlate with any measure of this study.

### Experimental design and statistical analyses

#### Behavioral measures of the TFS coding

Each of the following behavioral measurements was conducted on a different day from the others to randomize the influence of factors that may be idiosyncratic to a specific test day/session. A single lab visit contained only one behavioral measurement to reduce the impact of cognitive fatigue from hour-long experiments.

##### FM detection thresholds

To obtain monaural TFS sensitivity, FM thresholds were measured separately in each ear, using a weighted (3:1) one-down-one-up (Kaernbach, 1991), two-alternatives-forced-choice (2AFC) adaptive procedure. The stimulus in the target interval was a 500-ms-long 500-Hz tone with FM at a 2-Hz rate and variable depth. The reference interval was a 500-Hz pure tone. The interstimulus gap was 900 ms. The stimulus was ramped on and off with a rise/fall time of 5 ms to eliminate audible transitions. The stimulus level was 70 dB SPL. The subjects were instructed to press a button to indicate the interval containing the FM. Each measurement block was terminated after 11 reversals and the median of all the reversals from the adaptive procedure was extracted as the threshold. Four blocks of measurements were obtained in each ear from each subject. Except for an additional “demo” block to orient the participants before the formal testing, there was no further training. Sennheiser HDA 300 over-the-ear headphones were used for stimulus delivery. The slow FM rate of 2 Hz was chosen because it is thought that TFS cues are used to detect FM at rates below ∼10 Hz (Moore and Sek, 1996; Strelcyk and Dau, 2009). However, recent evidence suggests that this may not be the case (Whiteford et al., 2020). Nonetheless, given the large body of literature using and interpreting slow-FM detection as a measure of TFS sensitivity, we chose to include this in the battery of candidate measures.

##### ITD detection thresholds

To obtain a binaural measure of TFS sensitivity, we measured ITD detection thresholds using a three-down-one-up, 2AFC adaptive procedure. The stimulus consisted of two consecutive 400-ms-long, 500-Hz tone bursts with an ITD. The leading ear for the ITD was switched from the first burst to the second. The stimulus was ramped on and off with a rise/fall time of 20 ms to eliminate audible transitions and to reduce reliance on onset ITDs. The stimuli were presented at 70 dB SPL. Subjects were asked to report the direction of the jump (left-to-right or right-to-left) between the intervals through a button press. It was preferable to have subjects indicate the direction of change because absolute lateralization can be influenced by multiple factors (Moore and Sek, 2009). The threshold was defined as the geometric mean of the last nine reversals, and measured repeatedly across eight blocks, with a short break scheduled after the fourth block. Etymotic Research (ER-2) insert earphones were used for delivering the stimuli. A separate “demo” block was included before the experimental blocks to familiarize the subject with the task.

##### “Nonsensory” score

Because the main goal of the study is to evaluate candidate measures of TFS coding in naive subjects, i.e., individuals without extensive training/practice on the measured tasks, we anticipated that extraneous “nonsensory” variables may influence the measured thresholds. Accordingly, percent-incorrect scores on easy “catch” trials were calculated to quantify the subject’s engagement. Errors made in these catch trials likely reflect nonsensory factors such as lapses in attention, variations in motivation, alertness, etc., rather than the strength of sensory coding. For the FM detection task, trials with frequency deviations (modulation depths) >15 Hz were deemed to be catch trials, and the percent-incorrect scores were calculated for just these trials for each subject as an estimate of lapse rate. Similarly, the criterion for designating a trial as a “catch” trial for the ITD detection task was that the ITD exceeded 80 μs. The number of catch trials available varied from subject to subject because of the adaptive nature of the task. On average, the FM and ITD detection tasks included 3–10 catch trials per block. To mitigate the influence of extraneous variables such as engagement and motivation on the measured thresholds, a simple linear model was constructed with this nonsensory score as the sole predictor, and the residuals from the model were treated as “clean” thesholds and used in all analyses thereafter.

##### Supplementary amplitude modulation (AM) detection task

To further investigate the influence of nonsensory factors on behavioral measures in general, we conducted a supplementary experiment using a task that is unrelated to TFS processing, an AM detection task similar to the one used in Bharadwaj et al. (2015). A similar 2AFC procedure as in the FM and ITD detection threshold measurements was employed. The target was a 500-Hz, 75 dB SPL band of noise centered at 4 or 8 kHz, and amplitude modulated at 19 Hz. Two unmodulated tones, flanked at two equivalent rectangular bandwidths (ERBs; Glasberg and Moore, 1990; Moore, 1986) away from the center frequency, each at 75 dB SPL, were used to minimize off-frequency listening. The signal in the reference interval was statistically identical but unmodulated. Using a noise carrier helps eliminate spectral cues for the AM detection task (Viemeister, 1979). The threshold for the modulation depth detection was determined by an adaptive weighted one-up-one-down procedure (Kaernbach, 1991).

#### Electrophysiological measures of TFS coding

While behavioral measures directly assess perceptual sensitivity to TFS, they may also reflect common nonsensory factors such as attention and motivation. To dissociate TFS coding from nonsensory factors, we designed two passive EEG measures of TFS coding and compared them to individual behavioral measures. For EEG measurements, participants watched a silent, captioned video of their choice while passively listening to the auditory stimuli. EEG recordings were obtained using a 32-channel EEG system (Biosemi Active Two), while the stimuli were presented via ER-2 insert earphones.

##### General EEG setup and preprocessing procedures

The Biosemi EEG system employs active common-mode noise rejection using a pair of ground electrodes in a “driven-right leg” configuration (Metting van Rijn et al., 1990). EEG recordings were re-referenced to the average voltage across the two ear lobes. For cortical response analyses (EEG-ITD; see Cortical correlates of TFS-based ITD processing), the raw data were bandpass filtered from 1 to 50 Hz, whereas for subcortical responses (EEG-FFR; see FFR), raw data were filtered from 400 to 1300 Hz. The 400- to 1300-Hz bandpass filter eliminates artifacts from eye blinks. For the 1- to 50-Hz cortical data, ocular artifacts were removed using the signal-space projection technique (Uusitalo and Ilmoniemi, 1997). After the eye-blink correction, epochs with large voltage excursions (above 150 μV for cortical recordings; above 50 μV for subcortical recordings) were excluded to reduce movement artifacts. For both cortical and subcortical recordings, analyses focused on recordings from vertex electrodes (i.e., Fz and Cz channels).

##### Cortical correlates of TFS-based ITD processing

**Figure 1.**
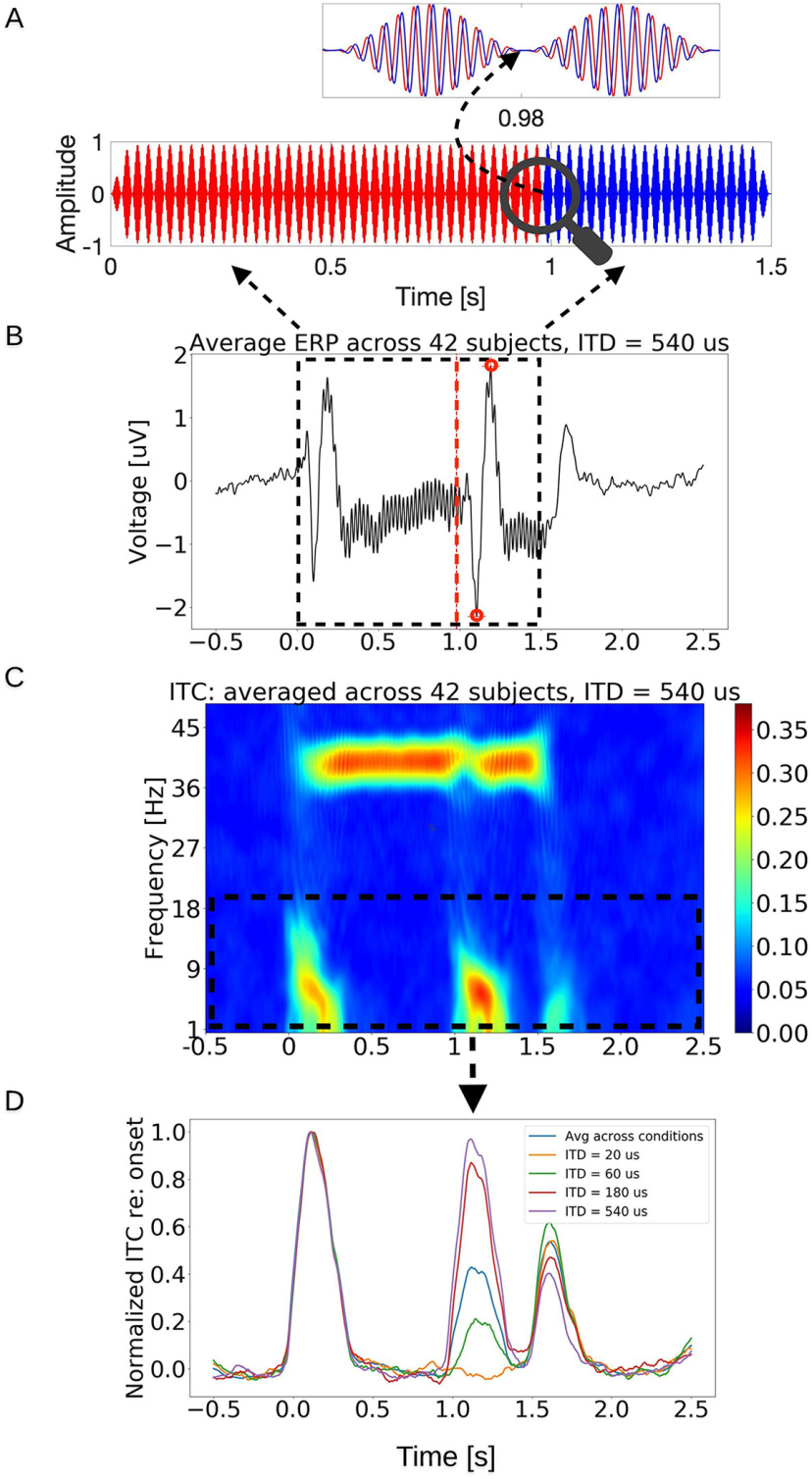
Stimulus paradigm and response from the EEG-TFS sensitivity measurement. ***A***, The stimulus is a 1.5-s-long, 500-Hz pure tone that is amplitude modulated at 40.8 Hz. The red color represents the sound in the right ear, whereas the blue stands for the sound in the left ear. In the figure, the stimulus in the right ear leads in time till 0.98 s (indicated by the red segment of zoomed-out view of the stimulus), after which the ITD shifts in polarity, i.e., the stimulus in the left ear takes the lead. The ITD jump occurs when the stimulus amplitude is zero to minimize the involvement of monaural cues (pointed out by the dashed arrow). ***B***, Averaged evoked response potential (ERP) from all trials across 42 subjects in “ITD = 540 μs” condition from Cz electrode. The red dashed line indicates where the ITD switched polarity, which resulted in N1 and P2 responses (denoted by red dots). ***C***, ITC spectrogram of the EEG response, averaged across 42 subjects, with the colormap indicating the ITC. Robust ASSRs can be seen around the AM frequency of 40.8 Hz. There are also salient responses time locked to the stimulus onset, offset, and importantly, to the ITD jump. ***D***, The average time course of the ITC for frequencies below 20 Hz is shown for each ITD jump condition. The response evoked by the shift in the ITD polarity increases monotonically with the size of the ITD jump, confirming that the response is parametrically modulated by TFS-based processing.

Cortical EEG was recorded in response to 70 dB SPL 500-Hz tones that were amplitude-modulated (100% depth) at 40.8 Hz. 40.8 Hz can elicit a strong auditory steady-state response (ASSR) in EEG recordings (Picton et al., 2003); this response was used here as a measure of recording quality (Fig. 1*C*). The stimulus duration was of 1.5 s. As with the behavioral measurement, the leading ear for the ITD switched 1 s into the trial. The direction of the ITD switch was randomized across trials. To minimize monaural cues, the ITD switch coincided with a trough of the 40.8 Hz modulation (Fig. 1*A*). This approach mirrors the method used in Papesh et al. (2017), where the stimulus switches between in-phase and out-of-phase states (phase shift of 180°). Our measurements involved ITD jumps of 20, 60, 180, or 540 μs in magnitude. The magnitude and direction of the ITD jump were randomized across trials. A total of 1200 trials were presented to the listener. The interstimulus interval was uniformly distributed between 500 and 600 ms. Besides amplitude and latency of the averaged evoked response across trials in each condition, we calculated the intertrial coherence (ITC), which quantifies the consistency in the phase of the evoked response components across trials. ITC of 0 indicates no phase locking (the response is dominated by background noise), and ITC of 1 indicates perfect phase-consistency across trials (no background noise added to the phase-locked response). Thus, the ITC is directly related to the signal-to-noise ratio of the evoked response (Bharadwaj and Shinn-Cunningham, 2014). The frequency band for ITC analysis was restricted to ∼1–20 Hz, because it is known that cortical transient-evoked responses primarily consist of low-frequency components, and because we sought to separate these responses from the 40.8-Hz ASSR response.

##### FFR

Subcortical FFRs were measured in response to tones in a forward-masking stimulus configuration (Verschooten and Joris, 2014). The stimuli consisted of three consecutive segments: a 500-Hz probe tone that was 100 ms long and at 75 dB SPL, a “forward-masker” tone of the same frequency and duration but at 85 dB SPL, and the same probe tone. A 50-ms silent gap was included between the first probe tone and forward-masker, but only a 1-ms gap was included between the forward-masker and the second probe tone. Each stimulus segment was ramped on and off over 5 ms to reduce audible transitions. The polarity of the stimulus was alternated across a total of 8000 trials. The 500-Hz component of differential response obtained across the two stimulus polarities reflects response components that are phase-locked to the TFS, whereas the summed 500-Hz response represents the response to the ENV. However, the TFS component can contain both preneural (e.g., cochlear microphonic; CM) as well as neural responses. Verschooten and Joris (2014) argued that the nonlinear residual obtained by subtracting the TFS response to the second probe tone from the TFS response to the first probe tone will isolate the neural component and suppress the approximately linear CM. This is because the forward masking of response to the second probe tone only masks the neural component, whereas the CM is intact. Owing to the inner-hair-cell rectification, the summed response across the two polarities also contains a component at twice the stimulus frequency (1000 Hz) that reflects physiological currents phase-locked to the TFS in the stimulus. Although TFS-related, whether this double-frequency response is purely neural as has been previously interpreted (Parthasarathy et al., 2020), or whether it includes preneural contributions is unknown. Thus, we considered two candidate subcortical correlates of TFS processing: (1) the 500-Hz component derived from the differential response across the two polarities of stimulus presentation, and (2) the 1000-Hz component derived from the summed response across two polarities of stimulus presentation.

#### Statistical analyses

Pearson correlations were calculated to illustrate simple associations between pairs of measurements. Statistical inference about behavior-physiology correlations was made using a multiple linear stepwise regression analysis by adding new potential predictors one by one to model the dependent variable. All reported significant associations met a false discovery rate criterion of 5% to control for multiple comparisons (Benjamini and Hochberg, 1995). Statistical analyses were performed using R (R Core Team, https://www.r-project.org/).

##### Code accessibility

Stimulus generation and data analyses were done using custom scripts. They can be accessed at: https://github.com/AgudemuBorjigin/stimulus-TFS,  https://github.com/AgudemuBorjigin/EEGAnalysis, and https://github.com/AgudemuBorjigin/BehaviorDataAnalysis.

## Results

### Nonsensory factors contribute to large individual differences in behavioral measures of TFS coding

Similar to previous reports of large individual differences in the AM and ENV-based ITD detection thresholds across NH listeners (Bharadwaj et al., 2015), both the FM and TFS-based ITD detection thresholds varied widely across our NH listeners. FM detection thresholds across 43 NH listeners ranged from 7 to 22 dB relative to 1 Hz [i.e., a frequency deviation (Fdev) of 2–13 Hz from 500 Hz]. ITD detection thresholds varied from 21 to 39 dB relative to 1 μs (i.e., 11–89 μs) across 37 NH listeners. These FM and ITD detection thresholds are shown along with the results from similar studies, in Figures 8 and 9, respectively, and were largely comparable.

**Figure 2.**
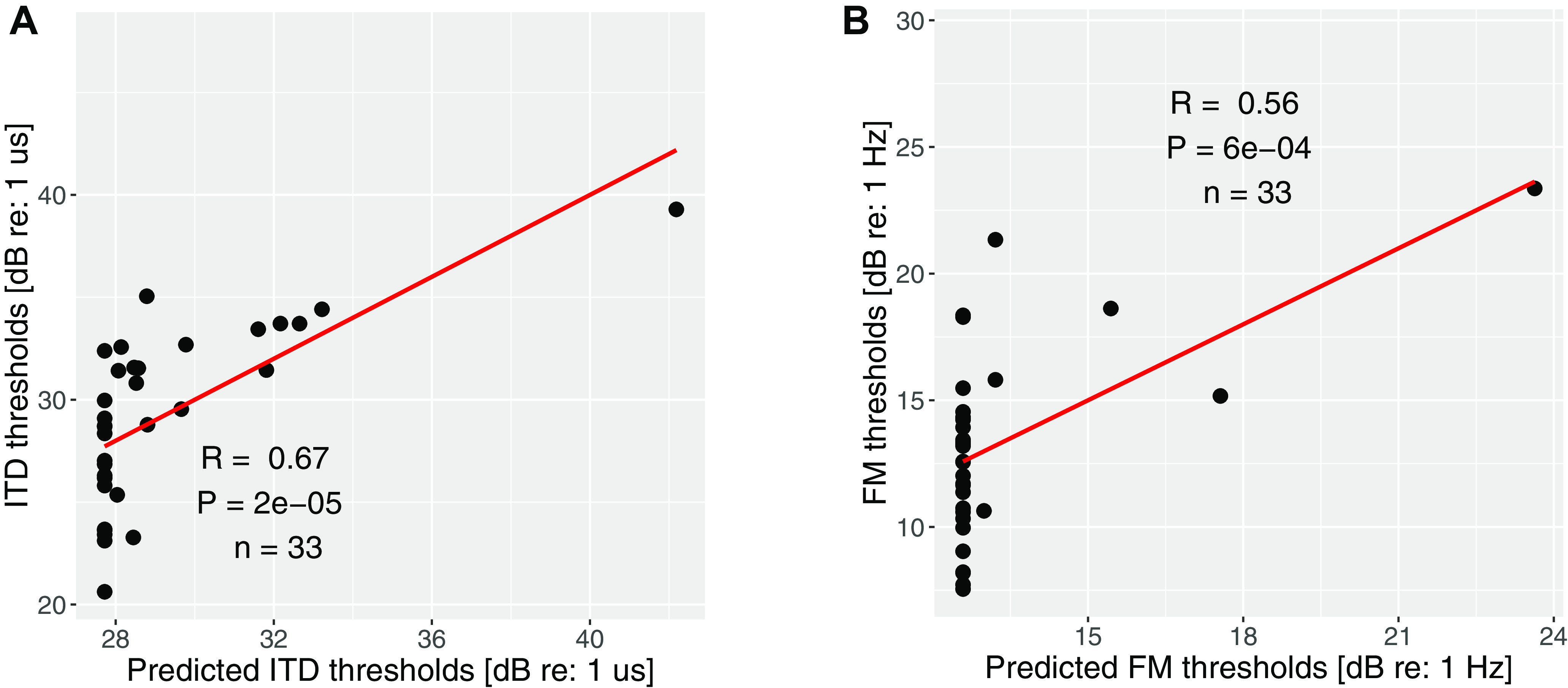
Measured versus predicted thresholds based on lapse rate. ***A***, Measured versus predicted ITD detection thresholds. ***B***, Measured versus predicted FM detection thresholds. The significant contribution of nonsensory factors is apparent, especially for the poorer performers.

Across listeners, neither FM (averaged across two ears) nor ITD thresholds (each averaged across repetitions) correlated with the audiograms (across-ear average of thresholds at 500 Hz; across-ear average of the mean thresholds at high frequencies: 4 and 8 kHz); however, the two measures were significantly correlated with each other in a simple linear regression analysis (*r* = 0.44, *p* = 0.01, *n* = 33). While the correlations may arise from individual differences in TFS coding, they can also reflect nonsensory factors such as attention, motivation, etc. To disambiguate these competing explanations, we assigned each listener a nonsensory score. When those scores were factored out from each measurement, the correlation between the monaural FM and binaural ITD thresholds dropped such that the association no longer met conventional statistical significance criteria (*R* = 0.31, *p* = 0.08, *n* = 33), suggesting that nonsensory factors play a large role in raw scores. Furthermore, when just the blocks with the largest (i.e., worst) FM and ITD thresholds for each subject were compared, considerably stronger correlations were observed (*r* = 0.6, *p* = 9e-4, *n* = 33), underscoring the involvement of nonsensory factors in behavioral measurements. Figure 2 shows the correlations between the measured and predicted thresholds solely based on the lapse rates (i.e., the nonsensory score). The involvement of nonsensory factors is evident, especially for the poorer performers.

**Figure 3.**
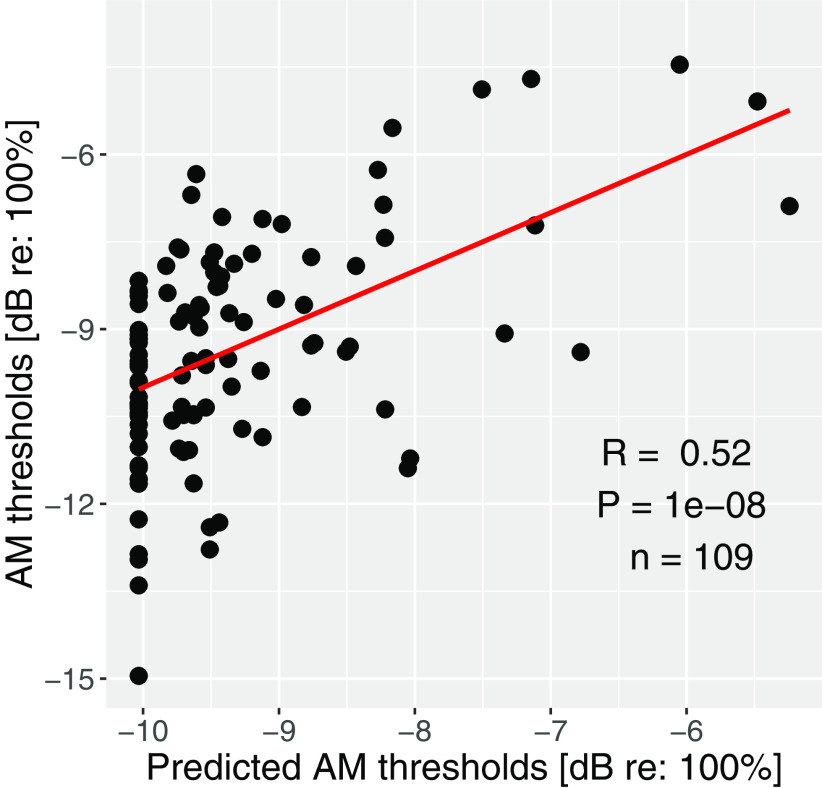
Measured versus predicted AM thresholds based on lapse rate. The thresholds are the average detection thresholds of AM tones at 4 and 8 kHz. The significant contribution of nonsensory factors is apparent.

To confirm the involvement of nonsensory factors in raw behavioral scores, a similar comparison of thresholds and lapse rates was conducted for the supplementary AM detection task. The predicted thresholds based on the nonsensory score significantly correlated with the measured AM thresholds (*R* = 0.52, *p* = 1e-8, *n* = 109; Fig. 3). This result indicates the significant weight of nonsensory factors, not only for FM and ITD detection measurements but behavioral measures in general.

### Raw electrophysiological TFS measures are strongly influenced by extraneous sources of variance

Two passive electrophysiological measurements were conducted to objectively evaluate individual TFS coding. Because passive electrophysiological measures are likely to be influenced by distinct extraneous factors (e.g., head size) compared with behavioral measures (e.g., motivation/engagement), these measurements provide a complementary window into individual TFS coding.

#### Candidate cortical correlates of TFS processing

**Figure 4.**
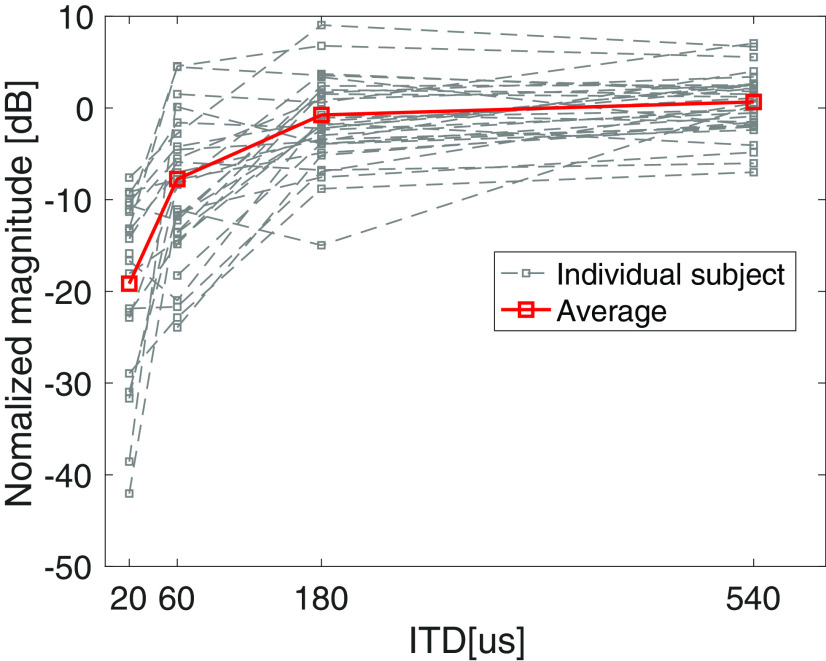
Individual EEG ITC (averaged under 20 Hz) values as a function of the jump size of the ITD. The ITC increases with the ITD for almost all subjects. Robust responses above noise floor are detected for most subjects for the “ITD = 180 μs” condition. Interestingly, individual differences present at 180 μs persist even at 540 μs despite the ITD jump being obviously perceptible and the response amplitude appearing to saturate.

Cortical responses evoked by the polarity shift of the ITD are quantified through the phase-locking strength shown in the phase-locking spectrograms (Fig. 1*C*). Clear responses to the onset, offset, and ITD jump are apparent in the low-frequency portion of the phase-locking spectrogram. The sustained ASSR is also clear around 40.8 Hz. The average response from 42 NH listeners shows monotonically increasing phase-locking strength of the ITD-evoked response across the ITD magnitudes (Fig. 1*D*), confirming that the response is indeed sensitive to TFS processing and the size of the ITD jump. Perhaps more important for the search of candidate TFS processing assays, large individual differences are apparent in the phase-locking strength across subjects (Fig. 4). Most subjects did not show a salient response for the 20-μs condition, and only about half showed robust responses for the 60-μs condition. Focusing therefore on the 180- and 540-μs conditions, the 180-μs condition is still part of the increasing slope of the response-versus-ITD-jump-size trend, but the response amplitude may have saturated for the 540 μs. Accordingly, we used each individual’s response for the 180-μs condition for comparison to behavior. Note that the ITD being referred to here is the size of the jump; for instance, for the 20-μs condition, the stimulus started with an ITD of 10 μs with one ear leading and jumped to the other side about halfway through the stimulus to end with a 10-μs ITD with the other ear leading.

Unfortunately, one striking aspect of the result in Figure 4 is that even at 540 μs, the individual differences that were present in the lower ITD conditions persist. The ITD jump is obviously perceptible at 540 μs, and the EEG response appears to be near saturation level for most individuals; this suggests that a significant portion of the individual differences in the magnitude of the cortical response arises from factors extraneous to TFS-based processing. Extraneous factors that may contribute include anatomic factors such as head size, and the geometry/orientation of the neural sources relative to the scalp sensors (Bharadwaj et al., 2019). Thus, although the cortical response to ITD jumps is indeed elicited and parametrically modulated by TFS-based processing, raw response amplitude metrics may be unsuitable for use as an individualized assay of TFS coding.

### Candidate subcortical correlates of TFS processing

**Figure 5.**
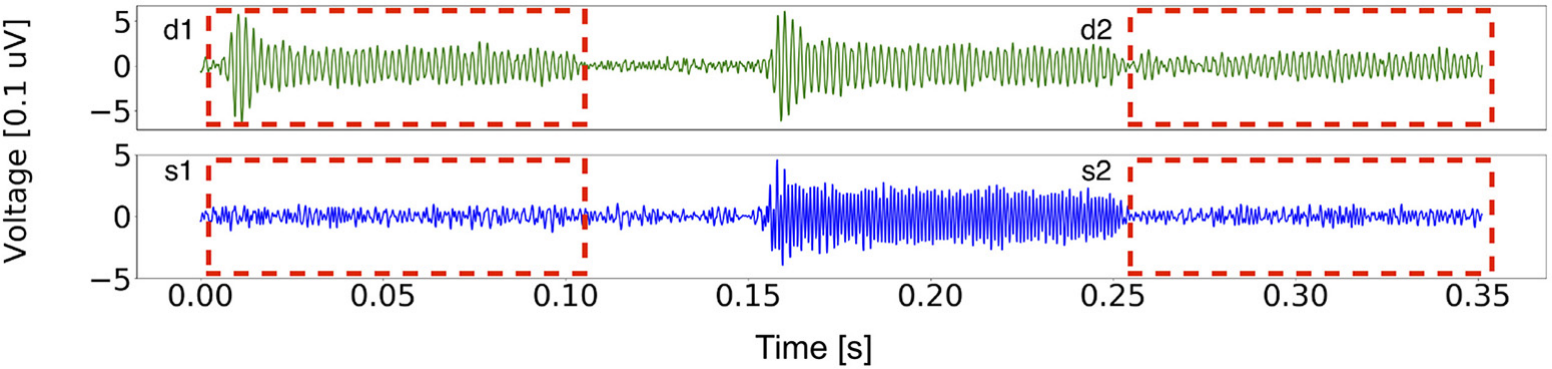
FFR to the probe-forward-masker-probe stimulus sequence for an individual subject. The top row (green trace) represents the differential response across two stimulus polarities, whereas the bottom row (blue trace) represents the summed response across two stimulus polarities. The first boxed segments in both rows (red, dashed box, labeled d1 or s1) reflect the raw response to the probe tone, which is likely a mixture of neural and preneural responses (e.g., CM), whereas the second boxed segments in both rows (red, dashed box, labeled d2 or s2) is the adapted response after forward masking. For d2 and s2, the preneural (e.g., CM) component is expected to be intact, whereas the neural response is attenuated by forward masking (because of a very short 1-ms gap). The forward masker only partially suppresses the responses, suggesting a strong preneural contribution to d1 and s1. The weaker residuals obtained by subtraction, i.e., (d1 – d2) and (s1 – s2) are likely purely neural.

Figure 5 shows an example FFR recording from a single individual in response to the stimulus sequence with a probe tone, a forward masker, and a second probe tone. The top row (green traces) shows the differential response across two stimulus polarities. This response to the probe tone (labeled “d1” in Fig. 5) tracks the 500-Hz TFS in the stimulus, but contains both preneural (e.g., CM) and neural components. Because forward masking is thought to arise from synaptic processing (Verschooten and Joris, 2014), the forward masker would be expected to only suppress the neural (i.e., postsynaptic) component of the response to the second probe tone, leaving the preneural component intact (labeled “d2” in Fig. 5). Thus, subtracting d2 from d1 should leave a purely neural response phase-locked to the TFS.

The bottom row in Figure 5, blue traces, shows the summed response across two polarities. Because of inner hair-cell rectification, this response contains a 1000-Hz component arising from the stimulus TFS (also see Materials and Methods). This 1000-Hz component in response to the probe (labeled “s1” in Fig. 5) has previously been interpreted as a neural response (Parthasarathy et al., 2020). If that were indeed the case, the forward-masker would considerably suppress the 1000-Hz component in response to the second probe (labeled “s2” in Fig. 5).

**Figure 6.**
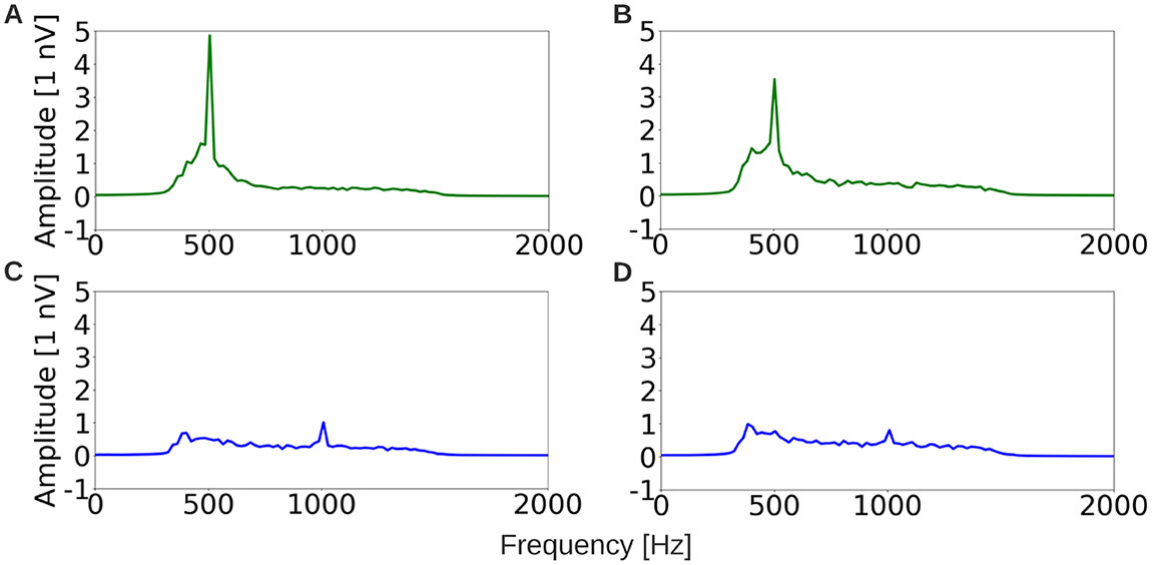
Frequency-domain representations of the d1 (***A***), d1–d2 (***B***), s1 (***C***), and s1–s2 (***D***) segments from Figure 5, but averaged across subjects. Forward masking partially attenuates both the 500-Hz component of d1 response, and the 1000-Hz component of the s1 response, suggesting that both responses reflect a mix of preneural and neural sources.

Figure 6 shows the average d1 (Fig. 6*A*), d1–d2 (Fig. 6*B*), s1 (Fig. 6*C*), and s1-s2 (Fig. 6*D*) response obtained across subjects, quantified in the frequency domain. It is evident from the reduced size of the (d1–d2) response compared with the d1 response, and the reduced size of the (s1–s2) response compared with the s1 response that, forward masker only has a partially suppressing effect. This provides evidence that both candidate TFS measures, the 500-Hz component from the difference across stimulus polarities, and the 1000-Hz component from the sum across stimulus polarities, have significant preneural contributions. This is in contrast to the previous interpretation that the component at double the tone frequency is purely neural (Parthasarathy et al., 2020).

These results indicate that a forward-masking paradigm will need to be employed to extract the purely neural “residual” response. Unfortunately, unlike transtympanic recordings that are difficult to perform (Verschooten et al., 2015), this residual is small and not readily measurable from all individual subjects. Thus, while subcortical envelope-following responses (EFRs) provide a robustly measurable correlate of envelope processing (Bharadwaj et al., 2015), tracking the TFS via FFRs are not promising, and not readily measured across all individuals despite our cohort being comprised of NH listeners.

### “Adjusted” behavioral and cortical measures are strongly correlated, likely reflecting TFS coding

Based on individual differences in the cortical amplitude measure persisting for the large-ITD-jump (540 μs) condition, we concluded that the amplitude measure of cortical phase-locking was dominated by extraneous variance, likely from anatomic factors. Thus, we focused our attention on the latency of the ITD-jump response, because the latency is expected to be unaffected by the scaling effects of individual anatomy. In particular, we extracted the latency of the cortical response to the 180-μs jump condition to avoid floor and ceiling effects. The latency was the mean of N1 and P2 latency (the latency is the time difference between the red dashed line and either N1 or P2 peak in Fig. 1*B*). The use of the latency metric was also motivated by the previous successful use of this EEG-latency measure to predict individual behavioral measures of spatial release from masking (Papesh et al., 2017). In addition to this latency metric, the slope of the cortical-response amplitude with increasing ITD-jump (i.e., the increase from the 60-μs condition to the 180-μs condition, divided by the 540-μs condition, in the ITC plot of Fig. 4) was extracted as a normalized measure of TFS processing that would mitigate the overall scaling influence of anatomic factors. This normalization was also motivated by the previous successful use of a similarly normalized electrophysiological measure in the context of modulation processing (Bharadwaj et al., 2015).

Both of these “adjusted” cortical measures exhibited significant correlations with behaviorally measured ITD thresholds. Specifically, individual differences in latency of the cortical ITD-jump response (for 180 μs) correlated with individual differences in the ITD detection thresholds (*R* = 0.35, *p* = 0.048, *n* = 32). The correlation improved when the behavioral scores were also adjusted to factor out the nonsensory score (*R* = 0.45, *p* = 0.01, *n* = 32). The slope metric from the cortical EEG response also correlated with ITD thresholds both with and without adjustments to the behavioral scores (*R* = 0.43, *p* = 0.021, *n* = 32, original ITD scores; *R* = 0.42, *p* = 0.028, with nonsensory score factored out). There were no significant brain-behavior correlations with “unadjusted” or “raw” metrics, such as the ITC amplitude of the ITD-evoked response, even after normalization by the ITC amplitude of the onset response.

With the subcortical measures, because results indicated a significant preneural contribution for both candidate TFS measures, and the residual neural component extracted from the forward-masking paradigm was not robustly measurable for many participants, we did not explore FFR-behavior associations in detail. A simple correlational analysis between the residual (d1–d2) 500-Hz response and ITD thresholds suggested that the correlations were not statistically distinguishable from zero (data not shown).

**Figure 7.**
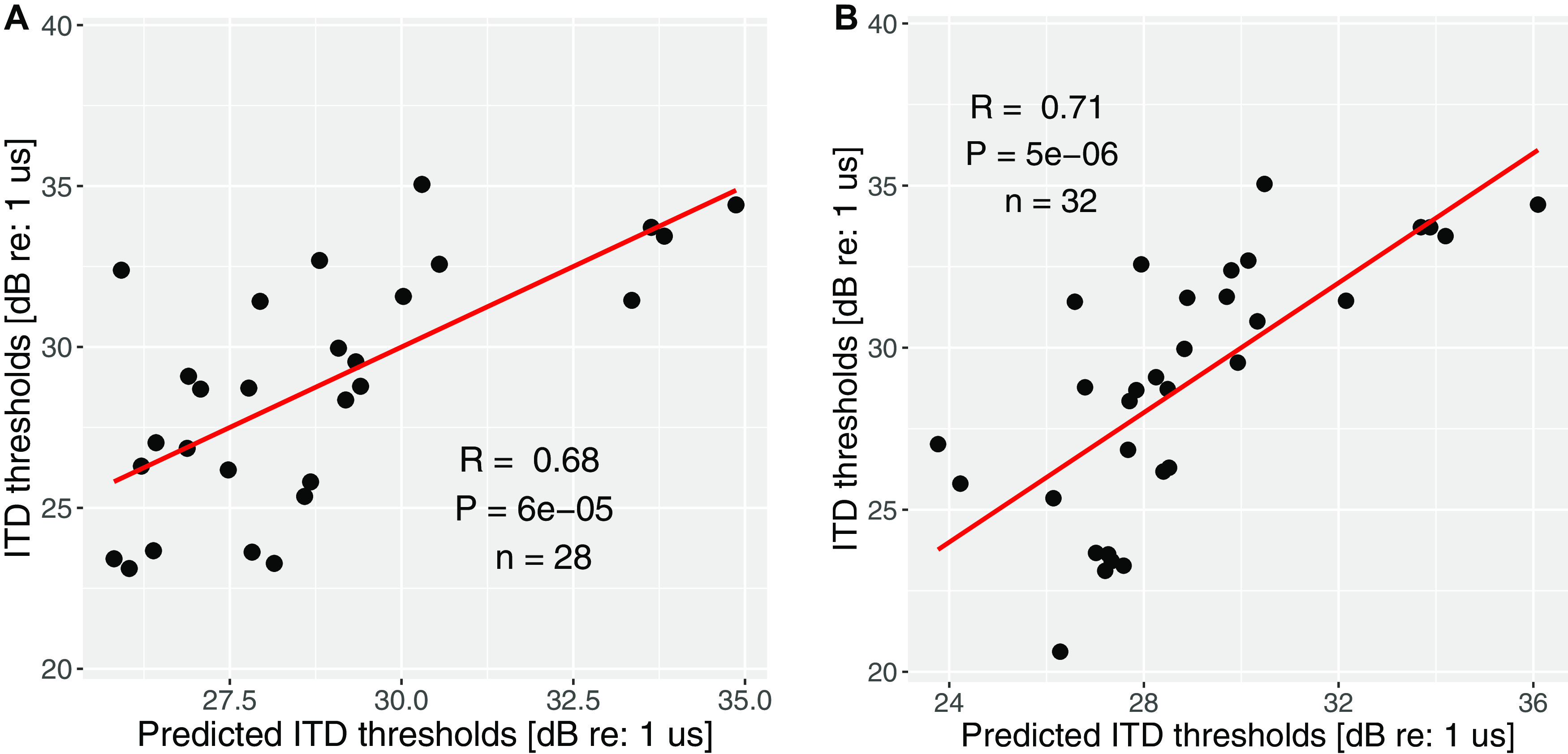
Model prediction of the ITD detection thresholds, based on the combination of lapse rate and slope (60- to 180-μs condition; ***A***), or the combination of lapse rate and EEG latency (***B***). Please refer to Table 1 for the variance explained by each factor.

A multiple linear regression model was used to predict ITD detection thresholds using both the nonsensory score, the EEG latency, as well as the EEG normalized slope metric (both from cortical ITD-jump response). The model could predict the behavioral ITD threshold well (Fig. 7) with the predictors together accounting for more than half of the variance observed in the behavioral thresholds (Table 1). We interpreted this result as suggesting that both “adjusted” behavioral scores, and electrophysiological latency or slope metrics in response to TFS-based binaural processing are promising candidate assays of TFS processing that may be suitable for use at the individual level.

**Table 1:** Model prediction of the behavioral ITD detection thresholds, with factors including the nonsensory score, EEG latency, and EEG slope

Predictor	Variance explained
Nonsensory score	37.48%
EEG latency	10.03%
EEG slope	9.28%
Explained	56.79%
Unexplained	43.21%

The variations accounted for by the nonsensory score are more than three times as by either one of the two EEG metrics. Together, more than half the variance can be explained.

**Figure 8.**
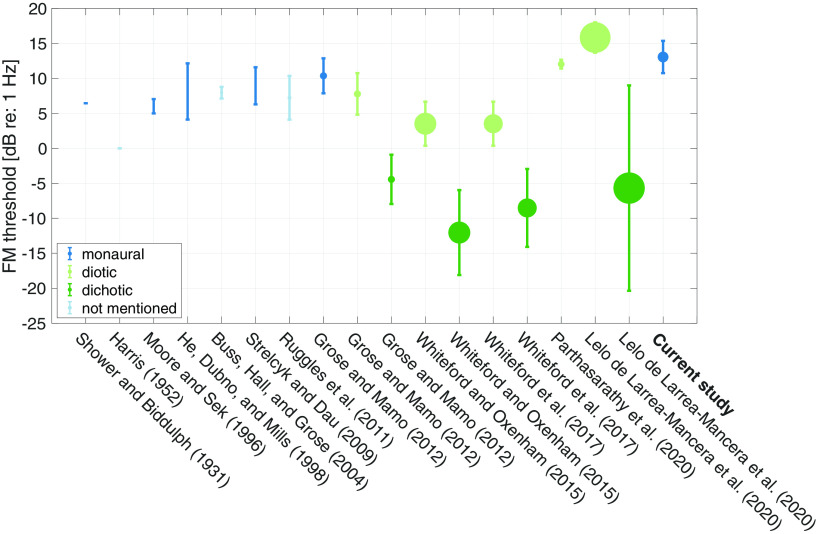
A sample of published reports of FM detection thresholds for comparison (Shower and Biddulph, 1931; Harris, 1952; Moore and Sek, 1996; He et al., 1998; Buss et al., 2004; Strelcyk and Dau, 2009; Ruggles et al., 2011; Grose and Mamo, 2012; Whiteford and Oxenham, 2015; Whiteford et al., 2017; Parthasarathy et al., 2020; Lelo de Larrea-Mancera et al., 2020). Error bar is 1 SD. The size of the dot represents the number of subjects (Whiteford and Oxenham (2015) has the most subjects; *N* = 100). Stimulus parameters such as stimulus level, carrier frequency, and modulation frequency in the cited studies are similar to those used in the current study, with slight differences (Strelcyk and Dau, 2009; Ruggles et al., 2011, used carrier at 750 Hz). Some threshold values are approximate from figures [e.g., mean and SD had to be estimated based on median and range in the box whisker plots in Whiteford and Oxenham (2015) and Whiteford et al. (2017)]. The mean and SD from the young and middle-aged group from Grose and Mamo (2012) were combined to generate a single data point. Some authors expressed the threshold in terms of Δ*F*/*F_c_*, where Δ*F* is frequency deviation, and *F_c_* is the carrier frequency. Moore and Sek (1996) used Δ*F* that was in two directions, i.e., peak-peak. Subjects from some studies were highly experienced in psychoacoustic tasks hence the thresholds were very low/good. Whiteford and Oxenham (2015) and Whiteford et al. (2017) obtained thresholds that fall in the lower end of the results of the current study from a very large number of subjects. This may be because their subjects were younger NH listeners and the stimuli were presented diotically and dichotically instead of monaurally.

**Figure 9.**
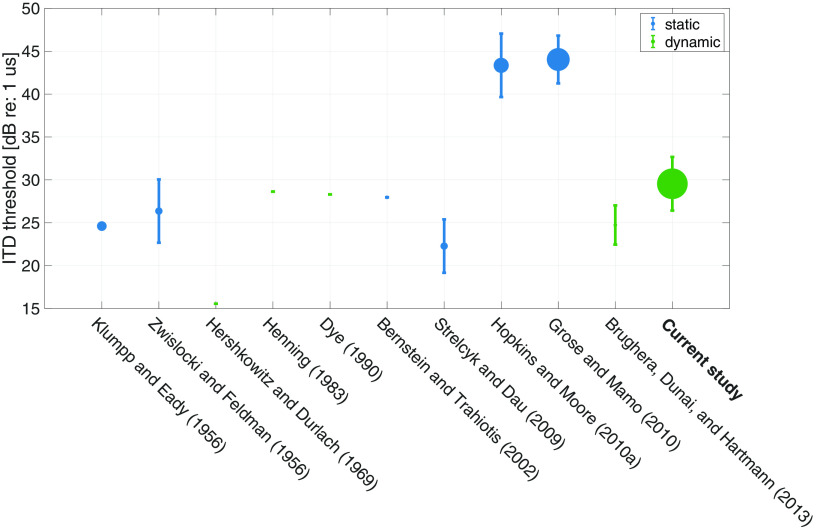
A sample of published reports of ITD detection thresholds for comparison (Klumpp and Eady, 1956; Zwicker, 1956; Hershkowitz and Durlach, 1969; Henning, 1983; Dye, 1990; Bernstein and Trahiotis, 2002; Strelcyk and Dau, 2009; Hopkins and Moore, 2010; Grose and Mamo, 2010; Brughera et al., 2013). Error bar is 1 SD. The size of the dot represents the number of subjects (the current study has the most subjects; *N* = 36). Stimulus parameters such as level and carrier frequency in the cited studies are similar to those used in the current study, with slight differences [Strelcyk and Dau (2009) used carrier at 750 Hz]. Note that some threshold values were extracted approximately from figures rather than direct numerical reports. Some of the studies used stimuli with the leading ear switching from one side to the other (labeled “dynamic,” marked in green color), whereas others presented an ITD only in the target intervals, with the reference being the midline (labeled “static,” marked in blue color). Note that the values from Hershkowitz and Durlach (1969) and Brughera et al. (2013) were halved since the authors used *ITD*/2 in each interval. The mean and SD from young and middle-aged cohort from Grose and Mamo (2010) were combined to generate a single data point. Subjects from some studies were highly experienced in psychoacoustic tasks.

## Discussion

In the present study, we sought to identify viable assays that can index the fidelity of TFS processing at the individual subject level. To obtain insight into whether individual differences in various candidate measures reflected TFS-based processing or extraneous factors, we compared individual differences in behavioral scores across FM and ITD detection tasks to differences in cortical and subcortical EEG-based measures. Results revealed the strong influence of extraneous factors on both behavioral scores and amplitude-based EEG metrics.

With behavioral measures, nonsensory factors quantified using the lapse rate in catch trials, could account for a third of the variance across individuals. Although previous work has explored a range of behavioral TFS measures (Moore and Sek, 2009; Hopkins and Moore, 2010; Sęk and Moore, 2012), the results from the present study underscore the importance of adjusting raw behavioral scores to reduce the impact of nonsensory factors. Indeed, although raw FM and ITD measures correlated significantly with each other, similar to the correlation between monaural AM detection and binaural envelope-ITD thresholds (Bharadwaj et al., 2015), this was driven in part by nonsensory factors. Because phase-locking to the TFS is essential for low-frequency ITD processing (Yin and Chan, 1990), it is plausible that ITD detection thresholds can provide an index of TFS sensitivity. On the other hand, whether FM detection relies on TFS coding has been controversial because of the possible role of recovered ENV cues that result from cochlear filtering of FM stimuli; indeed, FM stimuli lead to perceptible out-of-phase ENV fluctuations at cochlear places tuned to frequencies just above and below the FM carrier (Whiteford and Oxenham, 2015; Whiteford et al., 2017). Whiteford et al. (2020) extensively tested the role of place coding in FM detection and found that place coding by itself can account for the observed variations in FM sensitivity across all carrier frequencies and modulation rates. This finding is in contrast to the widely accepted view of the utilization of time coding in the detection of slow-rate FM (Moore and Sek, 1996; Strelcyk and Dau, 2009; Parthasarathy et al., 2020). Together with our finding that nonsensory factors influence raw behavioral scores, this uncertainty about the link between TFS coding and FM detection calls into question the previous use of FM detection scores as a correlate of TFS processing. In contrast, unambiguous theoretical links can be made between ITD detection and TFS coding, suggesting that once ITD thresholds are adjusted to reduce the influence of nonsensory scores, they may serve as a useful metric of TFS processing. This was corroborated by our finding that passive EEG measures, when combined with nonsensory scores, can account for more than half of the variance in ITD thresholds. Here, we used lapse rates in the catch trials to obtain a correlate of nonsensory factors. Alternately, a surrogate behavioral task that does not rely on TFS coding (e.g., interaural level difference sensitivity) may also be used to adjust ITD thresholds with similar benefits.

Another key finding from the present study is that although passive EEG measurements can potentially reflect TFS-based processing objectively, they too are susceptible to the influence of extraneous factors. Indeed, consistent with the interpretation that individual anatomic factors can have a scaling influence on response amplitudes, we found that cortical responses phase-locked to ITD changes showed large individual differences even for a large ITD jump (540 μs) where the response amplitude was near saturation for most individuals. Therefore, we argued that the evoked-response latency and/or percent growth/slope metrics may be better assays of TFS processing. Accordingly, latency and slope metrics showed significant correlations with behavioral ITD detection thresholds. For candidate subcortical FFR-based measures of TFS processing, our results showed that preneural physiological currents (CM, inner hair-cell currents) contribute significantly to the measure, thus complicating their applicability. Indeed, brainstem response measures from individuals with compromised inner hair-cell synaptic transmission show that preneural transduction currents can contribute to the measured response (Santarelli et al., 2009). Moreover, when employing a forward-masking-based design to isolate the neural component of the FFR, the resulting signal is relatively weak even in our NH cohort. This result from non-invasive ear-canal recordings is in contrast to neurophonic measurements from the auditory nerve (Snyder and Schreiner, 1985) or round window (Henry, 1995) from animals, or FFR measurements from humans using transtympanic electrodes where the forward-masking design has been used successfully (Verschooten et al., 2018). Although FFRs have previously been used as a putative correlate of TFS-based processing (Parthasarathy et al., 2020), our results suggest that additional experiments are needed to clarify the interpretation of those results.

Our finding that the subcortical FFR may be a poor correlate of neural TFS processing is in contrast to previous results suggesting that subcortical EFRs are correlated with behavioral measures of ENV processing. For example, Bharadwaj et al. (2015) showed that the AM detection thresholds and ENV-based ITD thresholds correlated strongly with normalized EFR-based metrics. This is likely both because EFR measurements more readily exclude preneural contributions (which primarily track the TFS), and because Bharadwaj et al. (2015) obtained asymptotic behavioral scores from a large number of trials (1200–1500 trials) from trained subjects. Indeed, with naive subjects in this study, an AM detection task similar to the one used in Bharadwaj et al. (2015) also showed a strong influence of nonsensory factors.

In summary, the present study examined various candidate assays for quantifying TFS processing at the individual subject level. These included behavioral FM and ITD detection thresholds, and EEG-based cortical and subcortical physiological measures. Among these, our experiments suggest that the latency of cortical responses to ITD jumps, normalized cortical response amplitude (i.e., percent growth/slope), and “adjusted” ITD thresholds may all be useful. Indeed, when a multiple linear regression model was constructed to predict behavioral ITD thresholds, the combination of the nonsensory score (lapse rate in catch trials), EEG latency, and slope measures could account for >50% of the variance across individuals. Our results are consistent with the findings by Papesh et al. (2017), who also found a correlation between ITD-evoked EEG latency and spatial-hearing outcomes such as spatial release from masking. Given that multiple candidate measures were explored to identify the most promising assays, future experiments should be conducted to independently confirm the efficacy of the assays endorsed by our results. The most promising assays rely on binaural TFS-based processing. Indeed, similarly to our results, steady-state cortical responses that track continuous interaural phase modulations have also been found to correlate with behavioral binaural sensitivity, further corroborating the potential utility of cortical binaural measures as electrophysiological assays of TFS processing (Undurraga et al., 2016; Koerner et al., 2020).

Reliable measures of TFS processing are critical for future investigations into the role of TFS in everyday hearing using intact speech-in-noise stimuli without vocoding manipulations. While sub-band vocoding can allow for independent manipulation of acoustic TFS and envelope cues, subsequent cochlear processing can confound these factors once again (Gilbert and Lorenzi, 2006; Swaminathan and Heinz, 2012). Furthermore, when both rate-place/ENV cues and TFS cues are redundant, vocoding experiments cannot provide insight into how they are perceptually weighted. The candidate TFS measures identified in the present study can help address these gaps.

## Synthesis

Reviewing Editor: Christine Portfors, Washington State University Decisions are customarily a result of the Reviewing Editor and the peer reviewers coming together and discussing their recommendations until a consensus is reached. When revisions are invited, a fact-based synthesis statement explaining their decision and outlining what is needed to prepare a revision will be listed below. The following reviewer(s) agreed to reveal their identity: NONE. Note: If this manuscript was transferred from JNeurosci and a decision was made to accept the manuscript without peer review, a brief statement to this effect will instead be what is listed below.

The reviewers agree that this is an important topic for study and the experiments are well done. The manuscript would be improved by providing more details about the methodology, a discussion about the effects of age, and some discussion of additional literature.

Reviewer 1:

I very much appreciate this manuscript. It was clearly written and provided important new information. I would suggest adding more information about the measures of non-sensory variables. This is something that other laboratories are likely to want to implement, and thus the number of catch trials and their timing in the experiments would be useful, as well as any other technical details necessary to implement this analysis. As it is, much is left unsaid, which could lead to another lab using a different approach and obtaining a different result.

In addition, I suggest that the data and approaches from two recent papers might be worth considering including. The first contains FM detection thresholds from 150 naive subjects, which could be used to supplement the data reported in Figure 8. https://asa.scitation.org/doi/full/10.1121/10.0002108

The second involves the use of an FFR measure that appears to be an assay of TFS based on binaural stimuli, the interaural phase modulation following response (IPM-FR). In our work, and in that of several others, the IPM-FR has been found to be related to behavioral measures of binaural sensitivity. This would be worth discussing in the section on potential electrophysiological assays of TFS.

https://www.frontiersin.org/articles/10.3389/fnins.2020.578566/full

Reviewer 2:

This work was designed to evaluate behavioral and electrophysiological measurement techniques that reflect TFS coding at the individual level and to explore attempts to minimize the influence of external factors on measures of TFS sensitivity. Methods of adjusting raw data improved the strength of brain-behavior correlations and helped to identify potential assays for quantification of TFS processing. This work highlights important findings regarding the effect of extraneous factors on behavioral and electrophysiological measures of TFS sensitivity. Additional details in the authors’ description of methodology, especially for the EEG measures, as well as a discussion on the effects of age on measures of TFS sensitivity would greatly improve the strength of this manuscript.

Pg. 4, Section 2.1:

-The authors should include more details about the audiometric inclusion and exclusion criteria used to select participants. It is mentioned later in the manuscript that all participants had normal hearing, but this should be explicitly stated and described in Section 2.1.

-In addition, given the wide 18-60 year age range of participants, it would be helpful for the reader if the authors could provide the distribution of ages of those who participated.

-Lines 91-93: the authors mention that not all participants were able to finish the full study. The authors should provide exact sample sizes either in the methods or results sections to display how many participants completed each measure, and how many participants were included to determine brain-behavior relationships.

-Pg. 6-8: Section on Electrophysiological TFS Measures

Additional EEG recording parameters should be included when describing methods for binaural ITD measure and FFR. For instance, what ground and reference electrodes were used? How long were the recording epochs? What thresholds were used for artifact rejection? What filters were used when processing the data?

Lines 153-161:

-The reader would benefit from more detailed information in the text about the stimulus used to elicit neural responses to the ITD cue. The authors mention that the ITD “switched halfway through the trial” but do not provide the length of the entire stimulus here. The author would have to look to the description of Figure 1 for this important information.

-How is the electrophysiological measure of ITD sensitivity (lines 153-161) different from that used by Papesh et al. (2017)?

-The reader would benefit from a description of what the authors are analyzing from this EEG response. For example, how were ITC values calculated? How should ITC values be interpreted in the context of this study? What is the significance of the frequency bands analyzed for the neural responses to the ITD shift? Some of this information is briefly described in the Results section on lines 232-235, but should be expanded on in more detail here.

-The authors should at least provide grand averaged time- domain response waveforms to depict this neural response in addition to the spectrogram shown in Figure 1.

-Lines 199-200: It has been well established through behavioral and electrophysiological studies that age likely impacts TFS processing even in normal hearing listeners. Given the wide 18-60 year age range of participants in the current study, it seems reasonable to assume that at least a portion of the variability in the behavioral and electrophysiological TFS measures described here may be due to differences in participant age. Can the authors comment on why they did not test for an effect of age on these responses or control for participant age in any of their statistical analyses?

-Line 207: When the authors mention that the FM and ITD thresholds were not “correlated with the audiograms", it is unclear what metric was used to quantify participants’ hearing sensitivity. This should be clearly described.

-Line 301: Did the authors also examine brain-behavior correlations with any of the “unadjusted” cortical measures? How did these relationships compare with those that used the adjusted measures?

-Lines 373-374: seems like a few words are missing from this sentence.
